# Mapping Hungarian procedure codes to SNOMED CT

**DOI:** 10.1186/s12874-023-02036-x

**Published:** 2023-10-18

**Authors:** Ágota Mészáros, Sándor Kovács, Tibor Héja, Zsolt Bagyura, Antal Zemplényi

**Affiliations:** 1 Department of Public Health, Semmelweis University, Budapest, Hungary; 2 Faculty of Pharmacy, Center for Health Technology Assessment and Pharmacoeconomic Research, University of Pécs, Pécs, Hungary; 3 Heart and Vascular Centre, Semmelweis University, Budapest, Hungary

**Keywords:** Real-world data, Data harmonization, Health data, Electronic medical record, Federated data network

## Abstract

**Background:**

Data harmonisation is essential in real-world data (RWD) research projects based on hospital information systems databases, as coding systems differ between countries. The Hungarian hospital information systems and the national claims database use internationally known diagnosis codes, but data on medical procedures are recorded using national codes. There is no simple or standard solution for mapping the national codes to a standard coding system. Our aim was to map the Hungarian procedure codes (OENO) to SNOMED CT as part of the European Health Data Evidence Network (EHDEN) project.

**Methods:**

We recruited 25 professionals from different specialties to manually map the procedure codes used between 2011 and 2021. A mapping protocol and training material were developed, results were regularly revised, and the challenges of mapping were recorded. Approximately 7% of the codes were mapped by more people in different specialties for validation purposes.

**Results:**

We mapped 4661 OENO codes to standard vocabularies, mostly SNOMED CT. We categorized the challenges into three main areas: semantic, matching, and methodological. Semantic refers to the occasionally unclear meaning of the OENO codes, matching to the different granularity and purpose of the OENO and SNOMED CT vocabularies. Lastly, methodological challenges were used to describe issues related to the design of the above-mentioned two vocabularies.

**Conclusions:**

The challenges and solutions presented here may help other researchers to design their process to map their national codes to standard vocabularies in order to achieve greater consistency in mapping results. Moreover, we believe that our work will allow for better use of RWD collected in Hungary in international research collaborations.

**Supplementary Information:**

The online version contains supplementary material available at 10.1186/s12874-023-02036-x.

## Introduction

Secondary use of routinely collected health data has become essential in healthcare research. Real-world data (RWD) are playing an increasingly important role in generating real-world evidence for regulatory decisions about marketing approval of drugs [1, 2] or in health technology assessments to support reimbursement decisions.

The main sources of RWD are claim databases and electronic medical records (EMRs). Although both data sources have the advantage of containing structured, easy-to-process information about diagnoses and procedures, their use has several challenges in international research collaboration. There are differences in the database structure, design and content [3]. In addition, multinational data collection and analysis is limited because of differences in coding systems across countries for which internationally valid mappings do not always exist [4].

The need for data harmonization has been recognized in the EU. The European Health Data Evidence Network (EHDEN) [4] and also the Data Analysis and Real World Interrogation Network (DARWIN EU) [5] adopt common data models and establish federated data networks for research collaboration. The open-source Observational and Medical Outcomes Partnerships Common Data Model (OMOP CDM) [6] standardizes the data structure, format, and terminology of datasets from various sources, vendors and countries. This enables the application of common analysis codes through a federated data network where only codes and aggregated results are shared but not the data [7].

In many countries medical conditions are coded using national versions of ICD-10. The recently published new version of the WHO International Classification of Diseases (ICD-11) will lead to much more meaningful and detailed coding of patient data in the future [8]. However, procedures are usually represented with country-specific codes. Procedure codes are influenced by different healthcare and reimbursement systems. Therefore, they tend to show more heterogeneity than expected. Thus, mapping local procedure codes to harmonized standards is necessary to facilitate research collaboration.

Institutions joining the EHDEN consortium’s network of data partners will adapt their local or national terminologies to the standards proposed by OMOP. In Hungary for the first time, two universities (Semmelweis University and the University of Pécs) joined one of EHDEN’s data partnership calls and mapped their EMR data to OMOP. As part of this work, the biggest challenge was the mapping of the Hungarian procedure codes (OENO) [9, 10], to international standards. Besides the procedure codes, other parts of the EMR (conditions, drugs, etc.) were also transformed to OMOP.

The Hungarian OENO codes were derived from the International Classification of Procedures in Medicine (ICPM) codes published by WHO in 1978 [11]. The development of the ICPM was practically stopped by the WHO in 1989 [12], so the Hungarian codes were no longer linked to it. Since then, several new codes have been added and the codes have been developed mainly to support the reimbursement of fee-for-service and diagnosis-related groups [13, 14]. As a result, the coding system is tailored to the medical practice and insurance system in Hungary and is not linked to other commonly used international standards.

Systematized Nomenclature of Medicine Clinical Terms (SNOMED CT), maintained by SNOMED International, is the most comprehensive, multilingual clinical healthcare terminology globally, already mapped to other international standards [15]. In the OMOP system SNOMED CT is the standard terminology for procedures. The current study aims to map the Hungarian procedure codes to standard OMOP concepts and to present the main challenges of mapping and how they could be overcome.

## Methods

### Data and data preparation

#### Source vocabulary

Around 5,500 inpatient and 3,100 outpatient procedure codes have been introduced in Hungary [9, 10], but some are not in use anymore mostly because the procedures have become outdated. To optimize and join the efforts of the universities participating in the project, we focused on mapping those codes that were recently in use at the institutions. We excluded those procedure codes from mapping that were not used by any of the two universities between 2011 and 2021.

The procedure codes’ descriptions were often too concise to judge what clinical activity they represented. Therefore, to find the appropriate matching concept in SNOMED CT, the codes were grouped by clinical specialties, assigning each code to those specialties that use the codes the most frequently. With in-depth knowledge about the codes and underlying procedures medical professionals from each clinical specialty group (23 in total) were asked to participate in the mapping exercise.

Multiple clinical specialties could use some of the codes. For validation purposes, these codes (approx. 7%) were allocated in two or three specialty groups for mapping. Then the results were checked to test the consistency of their mapping.

In order to support the prioritization of the mapping exercise the overall frequency, the frequency in outpatient departments and the frequency in inpatient departments were calculated and shared with those involved in the mapping.

#### Target vocabularies

The main target vocabulary was SNOMED CT [15] codes in the Procedure domain. The domains are the modalities of the code in the OMOP CDM. Since the OENO codes are used not only for medical interventions but also for diagnostic tests and medical devices, Observation, Measurement, and Device domains were also used for mapping. For some of the codes other standard vocabularies of OMOP CDM were found more suitable, such as Logical Observation Identifiers Names and Codes (LOINC) [16], RxNorm [17], and HemOnc [18]. Procedure descriptions in some cases include information on conditions, but these were not included in the target domains because we wanted to avoid having multiple, potentially contradictory, information on the diagnosis. The Hungarian diagnosis vocabulary (BNO) is used explicitly for coding diagnoses, therefore, that was considered as the primary source of information for diagnoses and mapped to SNOMED CT.

### Mapping process

The mapping project was carried out by a large research team (including PhD students, resident physicians and one specialist doctor). We developed a mapping protocol based on an Australian mapping guideline [19], Observational Health Data Sciences and Informatics (OHDSI) materials [20, 21] and with the support of an international medical code expert. For outpatient codes, we used the outpatient code rulebook [9], which contains coding rules and - in most cases - code definitions. We used these as a guide to understanding the content of the code. For inpatient codes, only the coding rules were available, no precise definitions for the codes were accessible. We pilot-tested the mapping protocol on 50 random codes with three core team members and refined it afterward. The mapping protocol can be found in the appendix (see Additional file 1).

**Fig. 1:**
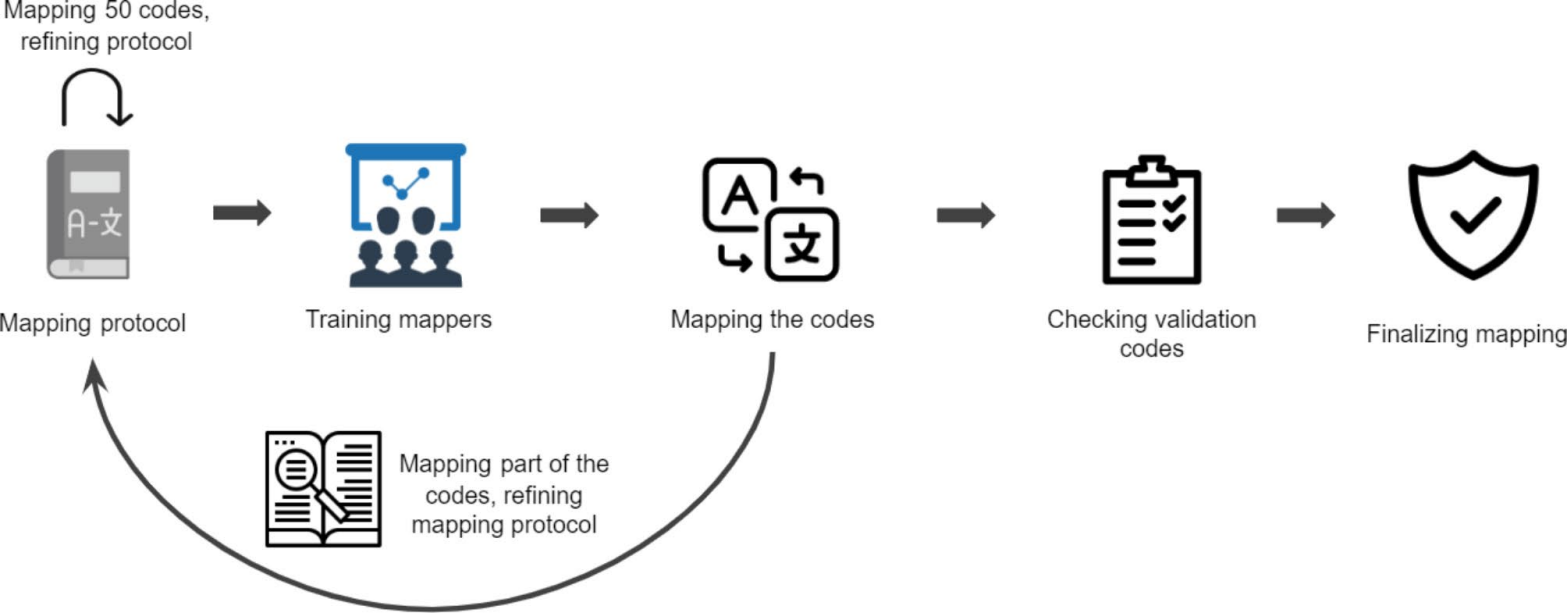
Outline of the mapping process

We held a training for the mappers before the start of the mapping. To test their knowledge and find out the mistakes and misunderstandings in the protocol and training material, first we asked the mappers to match 20–30 OENO codes as a pilot and send the results for review. Based on the pilot results, we held a meeting to explain the most common mistakes and how to overcome them and answered questions. As a next step, we asked the mappers to send back 30% of the codes assigned to them. Results were revised again and typical mistakes were used to develop the mapping rules further. Meetings were regularly held and there were opportunities for one-on-one discussions via phone, email, and video calls until every professional finished mapping the codes assigned to them. Then the selected codes were checked for validation. In case of differences, the more accurate mapping was chosen. The outline of the mapping process is shown in Fig. 1.

### Finding a match

When mapping from the source to the target vocabularies, we primarily aimed to find a target concept equivalent to the source in meaning. In these cases, we noted that this was an equivalent match. In the instances when there was no equivalent target concept, we mapped to a wider target concept and marked it as a wider match.

During the matching we had to decide whether to use only pre-coordinated concepts or post-coordinated expressions. Pre-coordinated concepts are usual concepts in one of the vocabularies, while in post-coordinated expressions, there is a relationship between more concepts; therefore, together they mean more. SNOMED CT is configured to make post-coordinated expressions, but in OMOP CDM they are not usable for procedures. There is no possibility to establish a relationship between two procedures or a procedure and an anatomic site. Therefore, we used only pre-coordinated concepts and mapped them to a wider concept in the absence of an equivalent concept.

## Results

### Results in numbers

The number of in- and outpatient procedure codes used by the two universities were 6049 in total. 558 laboratory test OENO codes were excluded because we did not map the laboratory procedure codes, just the laboratory measurement codes present in the EMR. Codes only used temporarily in a pilot project were excluded as well, total of 215 codes. From the OENO codes we mapped, the chemotherapeutic procedures (615 codes) were also mapped to OMOP CDM concepts – specifically to HaemOnc and RxNorm vocabularies – but with a different methodology, so they are not included here. This resulted in 4661 codes, of which twenty-three procedure code groups were created by specialties, each with 50–500 codes. These 4661 OENO codes were mapped to 6184 target codes, on average, one OENO code was mapped to 1.3 international codes, ranging from 0 target codes to up to 20 target codes, in rare cases.

**Fig. 2:**
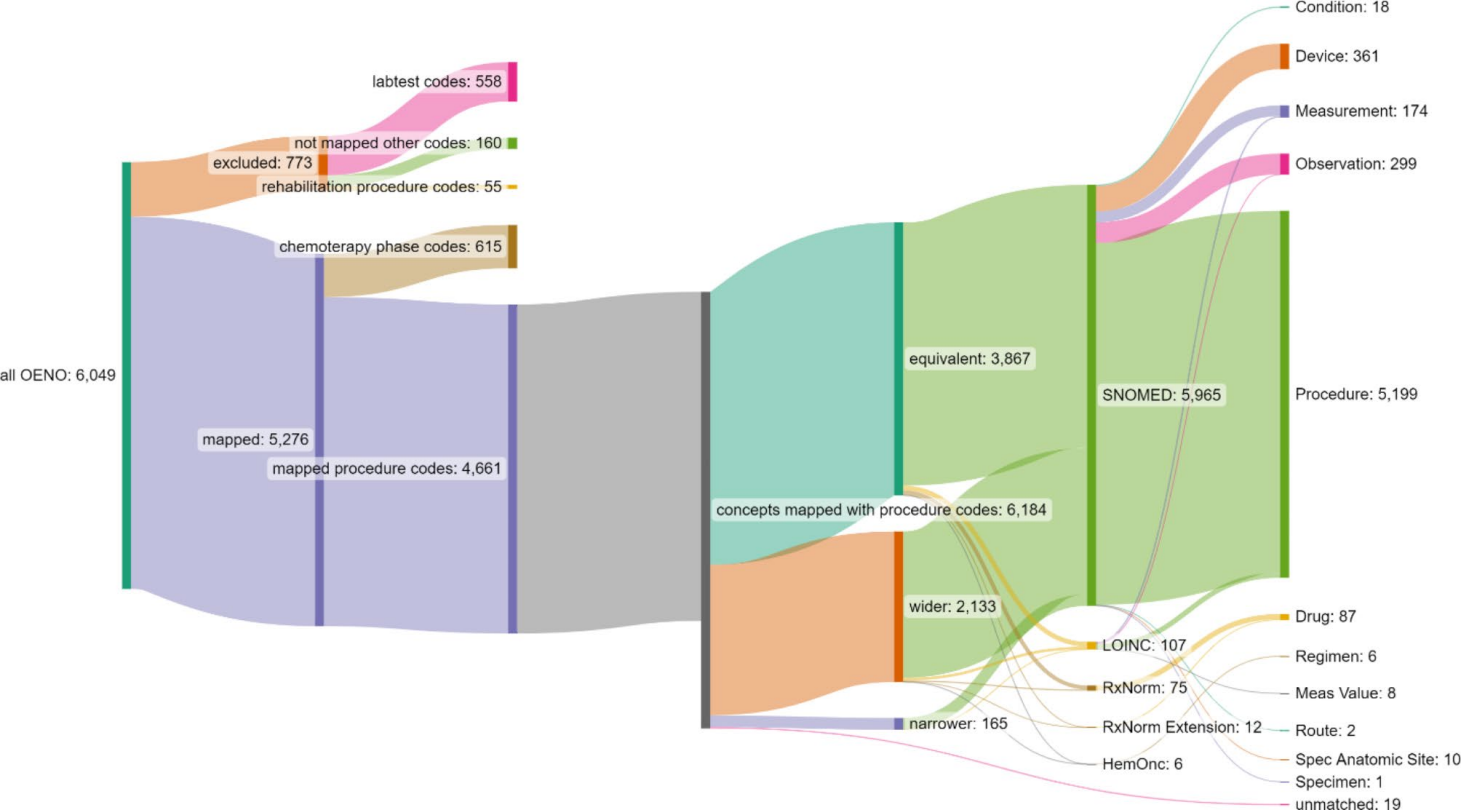
Vocabularies and domains to which the OENO codes have been mapped

Domain names [22, 23]: Drug: a biochemical substance ingested or otherwise introduced to the patient’s body; Measurement and its result Meas Value: structured values obtained through systematic and standardized testing of a patient or their sample; Observation: clinical facts about the patient; Procedure: activity performed in the provision of healthcare; Condition: disease or medical condition (stated as diagnosis, sign or symptom); Device: foreign physical object used for diagnostic or therapeutic purposes; Route: when and how a drug was given to the patient; Specimen: matter that is taken from patients for examination or analysis; Spec Anatomic Site: the anatomical site from which the specimen was taken.

Regarding the match type, 3867 (62.5%) was equivalent, 2133 (34.5%) was wider, 165 (2.7%) inexact or narrower, and 19 (0.3%) unmapped. From those unmapped codes 12 codes were excluded because they were not relevant medically, and 7 codes because we did not find any good match.

Out of the 431 number of validation codes, which were in more than one specialty group, 258 (60%) were mapped the same, 145 (34%) differently with equivalence in the meaning, and in 28 (6%) cases there was still some partial matching but with difference in the meaning.

Figure 2. shows all the OENO codes and the vocabularies, domains they were mapped to. It shows that even though majority of the codes were mapped to SNOMED CT procedure domain, a substantial amount of them were mapped to other domains and vocabularies, like HemOnc, LOINC and RxNorm.

### Challenges in mapping

One of the main challenges of the pre-processing process was to classify the codes into the appropriate specialty group. In a first step the codes were assigned to the specialty area where they were most frequently reported. This did not always correspond to the specialty actually performing the intervention, so these codes were manually reclassified to the appropriate specialty during the process.

**Fig. 3:**
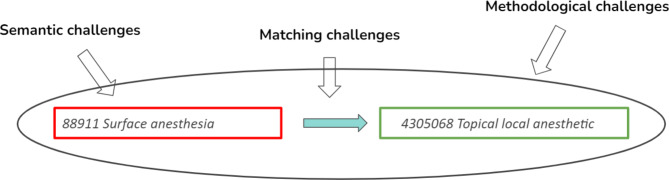
The three main challenges in mapping code from source to target vocabulary

The mapping of the OENO procedure codes was a bumpy ride, but we reached our destination – let’s hope no important chunk of information fell off the carriage. One of the bumps was a semantic challenge, interpreting the meaning of the OENO codes, which were ambiguous in some cases. The other bump was the matching itself, when there was no standard code that directly corresponded to the source code. The third bump was related to the different philosophies behind the two vocabularies, the methodological differences between the structure of the source and the target code vocabularies. Of course these challenges are intertwined: for example a semantic challenge poses a matching challenge in itself – not knowing the exact meaning of the code makes it difficult, if not impossible to find a right match. The relationship between the three main challenges is illustrated in Fig. 3. Examples of the challenges are given in Table 1. and the capital letters in the text refer to the examples listed in Table 1. For ease of interpretation the OENO codes presented here are the English translations of the Hungarian descriptions. Those in Latin were not translated.

**Table 1:** Examples of typical mapping cases and challenges

	Source (OENO) code and concept	Target code and concept
Example of a typical mapping case		
A)	16951 Choledochoscopy	4340102 Choledochoscopy
Semantic challenges		
B)	12115 Debunking visual examination	4108145 Optokinetic response
C)	TB143 Cystectomy	4323042 Excision of cyst
D)	42110 Screening of hip dislocation	46286410 Newborn and Infant Physical Examination Screening Programme, hip examination
Matching challenges		
E)	88916 Nerve block anesthesia on one finger or toe	4228073 Peripheral block anesthesia
		4178663 Anesthesia for procedure on extremity
F)	5396G Coronaria angioplastica on IVP branch (r. interventricularis posterior)	4184832 Coronary angioplasty
G)	5396N Coronaria angioplastica LM branch (main left coronary)	4184832 Coronary angioplasty
H)	2902V Amyloid staining to decide between AL and AA (as an additional examination)	4132406 Staining method
		4288616 Amyloid deposition
I)	99993 Additional point under 1 year of age	unmapped
Methodological challenges		
J)	98462 Treatment of gonorrhea with Rocephin	1777806 ceftriaxone (Ingredient)
K)	01050 Cochlear implant	4182413 Cochlear prosthesis (Device)
		4234743 Implantation of cochlear prosthetic device (Procedure)
L)	52072 Cochlear implantation	4182413 Cochlear prosthesis (Device)
		4234743 Implantation of cochlear prosthetic device (Procedure)
M)	57151 Vulvectomia unilateralis	4074152 Partial vulvectomy (Procedure)
N)	12655 Recognition and treatment of lipid metabolism disorder	43021492 Lipid disorder initial assessment
		44804677 Lipid disorder treatment started

The word in parentheses tells the domain of the code when relevant

#### Semantic challenges

In hierarchical code systems the codes “above” also known as parent concepts, and the codes “below”, children concepts, help to determine the meaning of the code. It is important to point out that the OENO code system is not hierarchical the codes are in a flat list. Usually, but not always, the codes that are close to each other are related to a similar specialty. Still, plenty of the codes were straightforward to understand based on their name and therefore to match, like in example A).

##### Cultural specificities

One type of semantic challenge arise from cultural specificity: when the code in question is not obvious in the meaning due to cultural and medical context or is understood only by a few specialists who use it. An example of this can be example B), the test for faking blindness or visual impairment, which is done when the doctor suspects that the patient’s vision is better than they indicate. Not surprisingly, there is no such code in OMOP CDM. The solution was to map a usual modality of this examination, the optokinetic response.

##### Ambiguous words in the name of the codes

There were terms that had more than one meaning and the relative brevity of the code names and the lack of hierarchy made it difficult to determine what they meant: as in example C) *TB143 Cystectomy* can mean *4029571 Bladder excision* or *4323042 Excision of cyst*.

One solution was to look at the OENO codes next to the code *(TB142 Retrograde root canal filling, TB144 Cystostomia, cysta-marsupialisatio)* which usually suggested the specialty using it. Furthermore, it was useful to search for synonyms by code name in the whole OENO code list, because codes with other meanings sometimes popped up *(55752 Hemicystectomia ves. urin., 55760 Cystectomia simplex ves. urin., 55761 Cystectomia totalis ves. urin.)*, therefore these meanings could be ruled out. In this example it was clear that the match is not *4029571 Bladder excision*, but rather *4323042 Excision of cyst*.

##### Unclear modality of the procedure

In some cases, it was not clear how procedure is performed from the name of the code itself, such as in the case of *42110 Screening of hip dislocation,* where it was not clear for the mappers, whether they should map *4085448 US scan of neonatal hip*, or *46286410 Newborn and Infant Physical Examination Screening Programme*, *hip examination*, or *4108627 Examination of hip joint*. Since this is an outpatient code we could look it up in the outpatient procedure codes rulebook. The rulebook stated that “Taking anamnesis, examination of the newborn (infant), physical examination of hips.“. This allowed us to select the appropriate match, as seen in example D). Although the outpatient rulebook was not always helpful, for example for inpatient codes. In these cases, we consulted specialists in the field to gain deeper insight into the underlying intervention.

#### Matching challenges

Matching challenges were usually present because of the different granularity of the vocabularies, and the purpose of the vocabularies: this is because the OENO’s aim was not to code the medical procedures in the most specific way possible but to code them specifically enough for financial purposes.

##### Location of the procedure

Quite a few codes do not specify the location of the procedure or have more than one location, such as E) *88916 Nerve block anesthesia on one finger or toe*. In SNOMED CT there is no code for finger OR toe anesthesia, only *4333956 Digital nerve block in hand* or *4337752 Local anesthetic digital nerve block in foot*. In this case, we could not map to both codes, so we searched for a less specific code and therefore lost some information: *4228073 Peripheral block anesthesia* and *4178663 Anesthesia for procedure on extremity*.

##### Granularity differences

The other end of the spectrum is when the OENO code system is a lot more granular than the OMOP CDM/SNOMED CT, and it is not possible to map the granularity of our codes to the standard codes, thus losing some information. The best examples are the codes between *5396G Coronaria angioplastica on IVP branch (r. interventricularis posterior)*, and *5396N Coronaria angioplastica LM branch (main left coronary)*, a total of eight codes, all of which can only be mapped to *4184832 Coronary angioplasty* as seen in example F) and G), because the lack of granularity in SNOMED CT. This led to the loss of some information in the original codes.

##### Reason of the procedure

OENO codes sometimes included not only the procedure itself but also the reason for the procedure in the code name, such as in H) *2902 V Amyloid staining to decide between AL and AA (as an additional examination)*. In SNOMED CT it is unlikely to find such codes where the procedure and the reason are found together in such detail. Thus, the closest codes we could find were the *4,132,406 Staining method* and *4,288,616 Amyloid deposition*, which again led to the loss of some potentially relevant information.

##### Codes used for reimbursement purposes only

There were some OENO codes that are used for reimbursement purposes only and do not represent any medical procedure or other relevant medical information. These codes were not mapped to the OMOP vocabulary. A good example is I) *99993 Additional point under 1 year of age*, which is used to indicate additional reimbursement claim due to the patient’s age. This information – the patient’s age – is already included in the EMR, and is loaded into OMOP CMD. In these cases the mappers were asked to leave it blank and not map anything to these codes.

#### Methodological challenges

##### Condition in procedure

Many OENO codes include conditions and diagnoses in their description, even though they are elements of a procedure code system. For example J) *98462 Treatment of gonorrhea with Rocephin*, this could be logically mapped to *1777806 ceftriaxone (Ingredient)* and *433417 Gonorrhea (Condition)*. But as mentioned in the Methods, we do not want to map conditions from OENO codes, so the correct match in this example is only *ceftriaxone*.

##### Device in procedure

The Hungarian claims codes include not only procedures, but in some cases also the devices used in the procedure or implanted in the patient. So when using a device, physicians had to code not only the procedure code but also the device code. It does not always happen, sometimes only one of them is coded, but it is not possible to do a *52072 Cochlear implantation* without using a *01050 Cochlear implant*, and vice versa. Therefore when mapping an OENO code for a device or device implantation, we sought to code both the device and the associated procedure so that no information is lost, as shown in examples K) and L).

##### Domains to map

In rare but important cases the domain (in OMOP CDM) gives an additional meaning to the concept, this may be particularly true for surgical concepts. In example M) the OENO code *57151 Vulvectomia unilateralis* means that the patient had a vulvectomy on one side at the date the procedure was recorded in the EMR. In SNOMED CT, there are two good matches to choose from: *4327505 Unilateral vulvectomy (Observation)* and *4074152 Partial vulvectomy (Procedure)*. The word in the brackets shows the domain and although the *Unilateral vulvectomy (Observation)* seems better, when the wording and domain are considered together, it may mean that the patient has had a vulvectomy on one side at some point and only the result is observed at the time of coding. Therefore, although less accurate translation, the *Partial vulvectomy (Procedure)* is a better match for the source procedure code.

##### One to many mapping – pre-coordinated or post-coordinated concepts

If an OENO code’s description contains a lot of additional information that makes it difficult to interpret, usually there is no single SNOMED CT code to match it. In these cases, such as in example N) *12655 Recognition and treatment of lipid metabolism disorder*, several codes can be found and a post-coordinated expression can be put together, such as *4159131 Dyslipidemia*, *4209828 Recognition* and *4079757 Treatment given*. It would be a fair match, if in OMOP CDM one could link the target concepts and use them as post-coordinated expressions. However, this is not possible for procedure codes, and the above codes do not make sense on their own. Therefore, in this case we looked for pre-coordinated concepts, *43021492 Lipid disorder initial assessment* and *44804677 Lipid disorder treatment started*, because they are good matches even on their own.

##### Hierarchy of target vocabulary

**Fig. 4:**
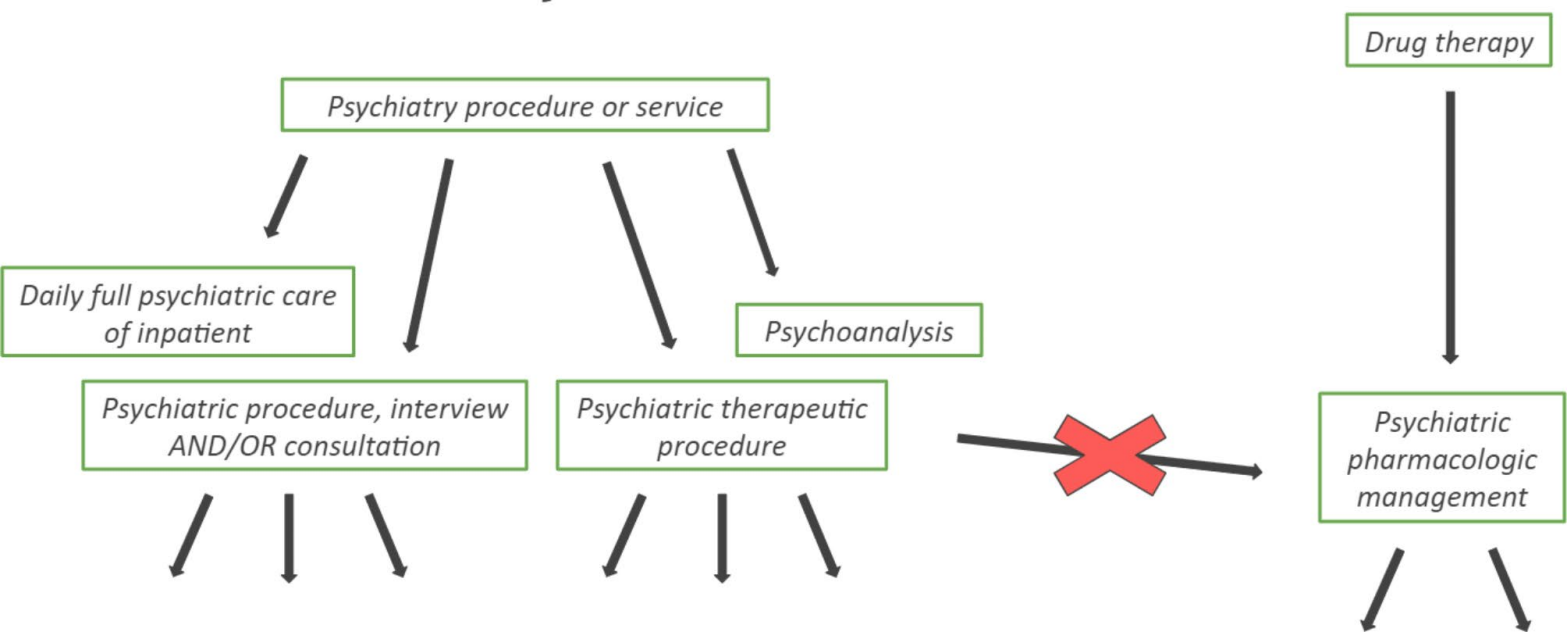
Partially connected hierarchy of some psychiatric procedures

Putting procedure concepts in a hierarchy – linked to conditions, devices, observations – can be a real challenge and as the OHDSI guidelines point out, the hierarchy of the procedure codes is not yet complete [24]. Yet, when searching for a target concept, a mapper looks at the parent and children concepts of a similar code to find a good match and because of the incomplete hierarchy, they might end up finding an inaccurate match. As an example, shown in Fig. 4., the code for “Psychiatric pharmacological management” is not part of the hierarchy of psychiatric procedures and therefore is not linked to “Psychiatric therapeutic procedure” neither.

## Discussion

We have developed a protocol and successfully mapped a total number of 4661 Hungarian procedure codes to SNOMED CT concepts. The mapping process itself was complex and required great attention to details with the potential for many errors and misunderstandings. In this study we presented the main challenges of mapping and how these could be overcome.

Matching challenges arose from the different granularity of the vocabularies and the way these vocabularies were created: SNOMED CT was established to be a standard vocabulary of medical terms, and OENO was developed mainly for financial purposes. The new codes and terms were added to facilitate reimbursement, so the aim was primarily to make them understandable to specialist doctors and medical coders. For this reason, it was sufficient if the term was financially accurate, but in many cases it was insignificant whether it was anatomically precise. For example, it was irrelevant whether the procedure (e.g., regional anesthesia) was performed on fingers or toes, the cost was generally the same.

Despite the validation codes were not perfectly the same in 40% of the cases, a manual check of the codes showed that in general both matches were plausible. It is just that sometimes, among the large number of possibilities, different mappers chose different ones, but both chosen concepts may be clinically sound. However, because the research cohorts are usually based on all the possible concepts of the same clinical event or condition [25], as long as the concept is clinically a good match, it will likely fall within the cohort definition. Still, it seems better for institutions in a country to use the same mapping rather than to each create their own to eliminate possible differences in mapping and to create a more reliable RWD for research.

### Similar studies

International literature on the subject is limited, but our study has drawn heavily on the experience of other workgroups.

A consortium of German university hospitals conducted a preliminary study on OMOP implementation but did not manually map procedure codes as this would require deep medical knowledge and resources [26] (2015). Later (2019) a feasibility study with 1000 procedure codes was conducted in Germany [27] using manual mapping, with challenges and findings similar to ours. (1) Some procedure concepts are generally only understood by specialists. (2) There is not always an equivalent match to the source concept, so there are match types expressing the equivalence: wider or equivalent. (3) These procedure codes are sometimes complemented with parameters important for billing such as vaguely described anatomic sites or numbers and these characteristics make it more difficult to map procedure (billing) codes to SNOMED CT, compared with cases where the anatomic sites are more precise and numbers are not present in the concept.

Besides the similarities, there were also some differences between our studies: they could not map all their codes with pre-coordinated SNOMED CT concepts, but they were able to use post-coordinated SNOMED CT expressions, which we did not use in our mapping, because they are not used in OMOP CDM. They did not map the devices that appear in the codes but in our case these were mapped and have their place in OMOP CDM. In their mapping they had 61% (n = 610) wider match and 34.2% (n = 342) equivalent, compared to our 34.5% (n = 2133) wider and 62.5% (n = 3867) equivalent. The difference may be due to the fact that the German billing code system has over 35,000 codes compared to our nearly 9000 codes, so their codes might be a lot more detailed, and therefore more difficult to map to an equivalent match. Although, using post-coordinated expressions might have made it easier to generate equivalent matches for them.

In South Korea, the reason for mapping a subset of their claim procedure codes was to explore ways to improve their own codes. They mapped 726 codes in five specialty areas, and in their mapping 41.9% (n = 304) of the codes were matched exactly and 40.7% (n = 295) partially, compared to our 62.5% (n = 3867) equivalent and 34.5% (n = 2133) wider [28]. Their findings show similarities to the problems with our national procedure codes: a hierarchy would be useful, and improving code labels would make the codes more understandable. Later, for research reasons, another subset, therapeutic and surgical procedures, was mapped to SNOMED CT from their claims procedure codes (2500 codes out of 9879) [29].

The main difference between our study and theirs was that they used post-coordinated concepts in half of the mappings similarly to Schulz et al. [27], i.e. they associated attributes and values to concepts. This is a useful way to explain complex source concepts, but is not compatible with OMOP CDM, so we could not use this method. Although the difficulties they encountered were similar to ours, such as the ambiguity of source codes and the need to clarify them. Moreover, they had similar issues with the nature of the claim codes: sometimes multiple procedure sites were included in one source code because it is usually indifferent for the healthcare payer where the procedure occurred. In their mapping, 63.6% of the target concepts were exact match to the source code, which is similar to ours; this may be due to the fact that we have a similar number of procedure codes, around 9000.

There are a few studies in the literature in which not procedure codes but other concepts, such as laboratory codes or emergency medical codes have been mapped to standard terminologies [30, 31]. These studies reported similar conclusions: the work was worthwhile and should be continued for good semantic interoperability and not all the national codes can be perfectly mapped to standard codes. For the German emergency codes they felt the need for national extension the SNOMED CT for unmapped terms, although for the Russian laboratory codes they concluded that those few unmapped codes are not so important. In our case, we were able to map nearly all the codes we wanted to map, but in quite a few cases only to a wider term, so there can be some lost information in the mapping, and solutions to this may improve the quality of standardized data in OMOP CDM.

### Limitations and strengths

Limitations of our work include that we did not map all the OENO codes due to resource constraints and because this mapping was sufficient to meet the needs of the project. In addition, despite our best efforts, there are still likely to be errors in the mapping, or cases where there may be a more precise match than we found. A further limitation was that some codes were very specific to a particular clinical area (e.g. early childhood development) and we were not able to involve the appropriate expert of the specialty, so there may be shortcomings in the mapping of these codes.

Strengths included the use of a well-developed mapping protocol and training material to ensure consistency in the process, the involvement of a wide range of specialists with practical and detailed clinical knowledge of the procedures, and the iterative process we used to review and validate mapping results to prevent errors in this demanding task.

## Conclusions

Future work should include refining the mapping, both methodologically but more importantly, looking for better matches where the match seems to be unclear. This could happen with the involvement of more specialists.

Mapping will need to be updated over time: new codes have already appeared and will continue to be introduced as medical diagnostic and therapeutic procedures evolve. For example, OENO codes for robot-assisted surgery have been added to the list since we finished our mapping. In order to ensure the mapping does not become outdated, regular update is needed to map the new codes from the national OENO vocabulary and possibly find a better match with the new standard codes.

To the best of our knowledge, there are no other studies on the mapping of Hungarian procedure codes to SNOMED CT, and the number of studies in the literature describing the process of mapping national procedure codes to SNOMED CT is also limited. Although the mapping process is different in each case, it is important to learn from other working groups. We hope that sharing our experience can help the community in this work and contribute to the great work of standardization of medical data in Europe and worldwide.

### Electronic supplementary material

Below is the link to the electronic supplementary material.

Supplementary Material 1

## Data Availability

The datasets used and/or analysed during the current study are available from the corresponding author on reasonable request.

## List of abbreviations

BNO: Hungarian diagnosis vocabulary
DARWIN: EU Data Analysis and Real World Interrogation Network
EHDEN: European Health Data Evidence Network
EMR: Electronic medical record
ICD: International Classification of Diseases
ICPM: International Classification of Procedures in Medicine
LOINC: Logical Observation Identifiers Names and Codes
OENO: Hungarian procedure codes vocabulary
OHDSI: Observational Health Data Sciences and Informatics
OMOP CDM: Observational and Medical Outcomes Partnerships Common Data Model
SNOMED CT: Systematized Nomenclature of Medicine Clinical Terms
RWD: Real-world data
