# Supplementary material for: Mapping Hungarian procedure codes to SNOMED CT
Source: BMC Med Res Methodol. 2023 Oct 18;23:240. doi: 10.1186/s12874-023-02036-x (PMC10585817; doi:10.1186/s12874-023-02036-x)
Supplement: Supplementary file 1 — Supplementary Material 1 [file 12874_2023_2036_MOESM1_ESM.docx]

**Mapping protocol 1.0**

**OENO codes to OMOP CDM**

**1. Before mapping**

**1.1 Purpose**

Purpose: The purpose of the map was the reuse of data, through joining EHDEN and OMOP-CDM, data reuse in Hungary and EU

Audience: EHDEN partner researchers

**1.2 Naming documents**

Parts of name of excel documents, between these parts there should be an underscore

- content of document, e.g. “trauma”
- which stage the document is in:
  - “rawdata” (downloaded data table, unprocessed)
  - “preprocessed” (broken down by specialty, unused codes removed)
  - “mapped” (mapped, unsure codes are flagged)
  - “final” (final, reviewed map)
- date, in “2021-04-28” format
- initials of the editor of the document e.g. "KS"

→ Example: OENO-trauma_preprocessed_2021-05-26_KS.xls

**1.3 Source and target terminologies, documentation**

OENO codes downloaded from Pulvita on 2021-05-14

OMOP CDM standard vocabularies downloaded from Athena in 2021-07-09

Mapping protocol 2023-02-01

**1.4 Personnel, mapping team**

The mapping team consisted of a core team from both the universities, IT specialists, medical doctors and PhD students in the medical field for mapping – in all important medical specialties, and outside advisors who we contacted regularly.

**1.5 Tools**

For mapping a Java-based software of OHDSI, Usagi (version 1.4.3) was used. Usagi has an in-built automated mapping feature, which matches the most likely target standard code to the source, based on term similarity approach[1]. As this automated tool works only in English the codes were translated with DeepL[2] and the translation was revised manually before loaded to Usagi.

Besides Usagi, the following tools were used in the mapping process:

- Athena vocabulary searching for target concepts,
- DeepL for translation hungarian code names,
- Microsoft Excel for preprocessing and reviewing the final map

**2. While mapping**

**2.1 Pre-processing source terms**

**2.1.1 Code groups**

Pre-processing file

| Outpatient occurrence 2012-2020 | Inpatient occurrence 2010-2020 | Source ID | Source description | Source DeepL translate |
| --- | --- | --- | --- | --- |
|  |  |  |  |  |
|  |  |  |  |  |
|  |  |  |  |  |

- Source description raw translations could be corrected before the coding or during the coding if needed.
- Coverage goal: 100% of the used codes from the last 11 years
- Downloaded from Pulvita, date: 2021.05.14.

**2.1.2. Checking the codes**

The codes and their frequencies were downloaded from Pulvita Health Data Warehouse[3] along with the type and speciality of the department they were coded in, with a timespan from 2010 (for outpatient codes from 2012) to 2020. Based on this information we filtered out the codes not used at our universities. We also deleted the codes starting with R, because they were made for a pilot program.

We checked all the codes if they are all 5 digit codes and if all the codes have a description.

**2.1.3. Grouping**

As well as the medical procedures behind them, there are inpatient, outpatient and universal codes. For grouping of the outpatient and universal codes a list[4] was used which specifies the owner of the code – from these 44 distinct specialities we made a grouping of 23 specialities. In the absence of a similar list for inpatient codes, we grouped them based on the department which used the code most frequently.

**2.2 Building the map**

Build map format

| Source ID | Source description | Source frequency | Source google translate | Target concept ID | Target description | Match type | Mapper | Status | Comment |
| --- | --- | --- | --- | --- | --- | --- | --- | --- | --- |
|  |  |  |  |  |  |  |  |  |  |
|  |  |  |  |  |  |  |  |  |  |
|  |  |  |  |  |  |  |  |  |  |

**2.2.1 Reference set**

For joining OMOP CDM, we needed to map the codes only to standard codes.

The main vocabulary and domain of the target concepts was SNOMED CT vocabulary and Procedure domain.

When the code itself was not so much a procedure, other domains or vocabularies could be used.

- drug (RxNorm)

*Addition of Cardioxan (dexrazoxan) to chemotherapy for haematological diseases --> 42736 dexrazoxane (****Drug****, RxNorm)*

- measurement (SNOMED)

*Abdomen circumference --> 396552003 Abdominal circumference (Measurement, SNOMED)*

- observation (SNOMED)

*Auditory training for cochlear implantation --> 108250004 Hearing therapy AND/OR auditory rehabilitation (****Procedure****, SNOMED) és 449840001 Cochlear prosthesis in situ (****Observation****, SNOMED)*

- device (SNOMED): our purpose was not to lose information neither on the device, nor the procedure/observation. Therefore our aim was to map a device and an observation/procedure as well to the source codes containing devices.

*Artificial lens --> 313236002 Intraocular lens implant (****Device****, SNOMED) and 69724002 Implantation of prosthetic intraocular lens (****Procedure,*** *SNOMED)*

- clinical finding, condition, spec anatomic site, specimen: do not use for the mapping

**2.2.2 Automated searching, how to chose**

The following figure[5], made by OHDSI, shows how to choose a target concept while mapping:


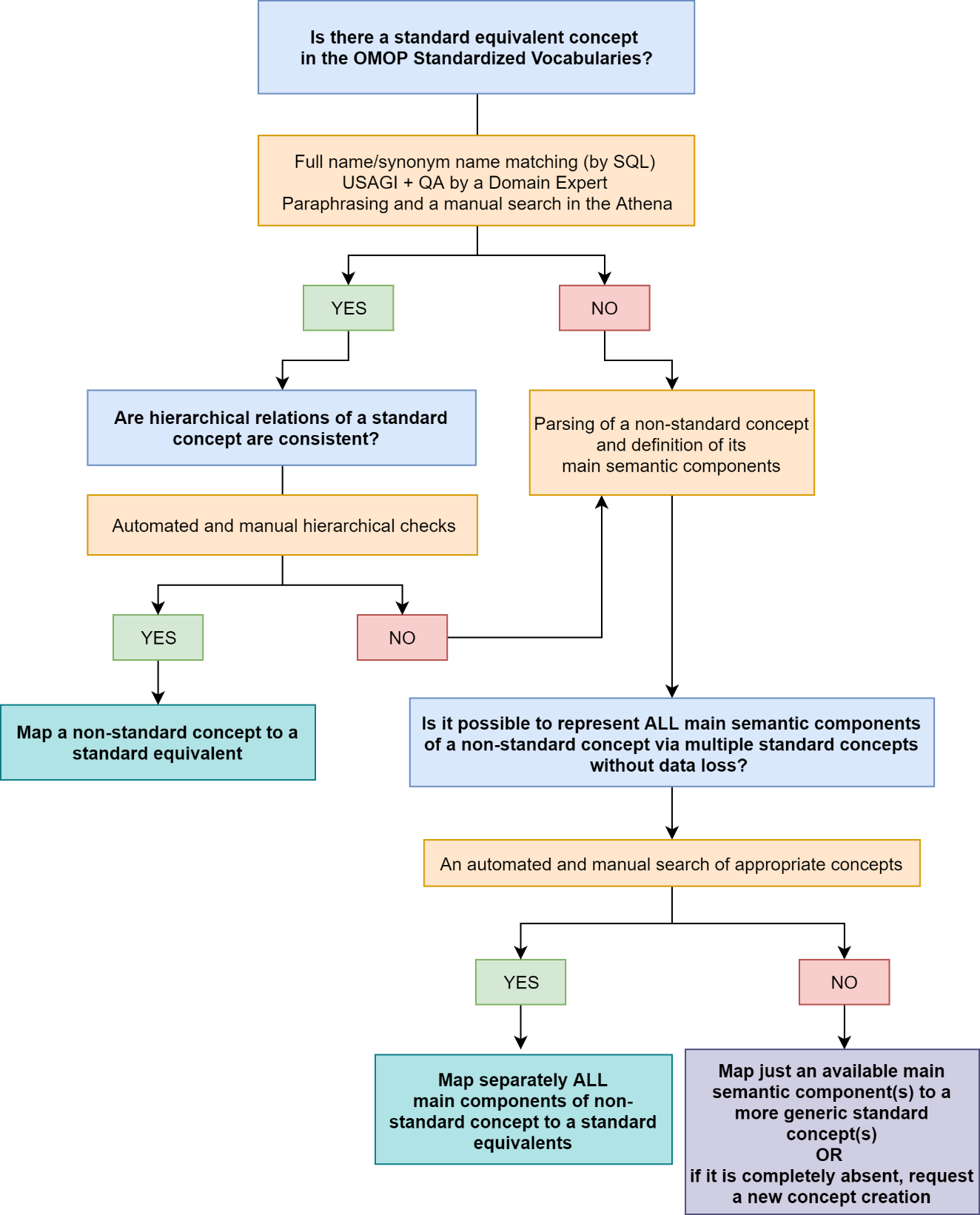


**Figure A1: Flowchart of the mapping process**

Mapping process:

1. In case of being unsure of the meaning of the source code:

- google search or google image search
- in case of outpatient codes the outpatient code rulebook can help with the longer descriptions, or even change the meaning a little [6]
- For inpatient codes maybe the inpatient rulebook can help [7], but it is not as useful as the outpatient one

1. First it is good to look at the automap suggested matches:

- if at first you see that the source term doesn't make sense, you can manually type a better translation into the query
- the good target concept may be somewhere later in the automap suggested matches
- if you are not sure in the meaning of your target concept, it is good to google search or google image search the target concept

*Example: Low-resistance sound prosthesis --> 360106001 Prosthetic voice box (Device, SNOMED)
→ similar, but not good match: 705372008 Voice-production device (Device, SNOMED)*, it is easily visible even on the google image search

1. Hierarchy: parent-children concepts (Alt+C in Usagi)

- If it's not clear if it's good a match, then look at the parent and children concepts of the closest matches, you can choose between them, or even "siblings" of a similar match (relevant parent concept’s children concepts)
- Before you approve a code, be sure to look at the hierarchy, there are some cases of where this alone will indicate that the target concept is not actually a good match

*Example: Low-resistance sound prosthesis --> 360106001 Prosthetic voice box (Device, SNOMED)
→ similar, but not good match: 272264002 Speech aid (Device, SNOMED),* because its parent is *Intraoral Device*, and the original OENO is a laryngeal replacement device

1. If a source concept contains several terms and there seem to be no suitable target concept

- The best solution is to find one target concept as a match
- If not, two or more codes can be mapped to it, one for each conceptual part
- it is advisable to write the terms in the query one by one
- Sometimes even more concepts can not describe the source concept fully

*Auditory training for cochlear implantation --> 108250004 Hearing therapy AND/OR auditory rehabilitation (Procedure, SNOMED) and 449840001 Cochlear prosthesis in situ (Observation, SNOMED)*

1. If there is no target concept that accurately describes the source

- a broader concept match is fine (or if the code can be split into several concept parts, a broader match described by several codes)
- But before finalizing the match as a broader concept, be sure to look at its children to make sure there's no matching one, and search for its synonyms

1. If there are several good matches, which one to choose

- Look at the domain that fits better (usually Procedure, for medicines Drug, etc.)

*Karnofsky-index --> 761870009 Assessment using KPS (Karnofsky Performance Status) (Procedure, SNOMED)* not 273546003 Karnofsky performance status (Measurement, SNOMED) or 761869008 KPS (Karnofsky Performance Status) score (Observation, SNOMED) or similarly named LOINC code

- If both domains are good, then look at the hierarchy of concepts and choose the one that has a more complete, more precise, more extended hierarchical network

*Hearing therapy for cochlear implantatum --> 108250004 Hearing therapy AND/OR auditory rehabilitation vs. 91193004 Hearing therapy,* the former has more cochlear implant rehabilitation concepts among its children, so it is the good choice (we cannot choose those concepts specifically because they are too specific), the latter does not have such a good hierarchical network

- If you still can't decide, choose the one with a shorter concept code (SNOMED International) as opposed to the one with a longer one (SNOMED UK/US Edition)[5]

1. Other: "not to be mapped" codes

- In some cases, the OENO code should not be mapped because it is redundant and does not contain relevant information (e.g. additional code for 3 years, additional code for patient care beyond 30 minutes) and therefore does not have a corresponding code
- In these cases remove the automap, approve the code so that there is nothing matched there

**2.2.3 Match type**

The time frame for finding the best match is 10 minutes. If you don't get a good match in that time, flag it and come back to it later.

You can find two types of matches for a code:

- Equivalent: target means the same as the source concept and is in the right place in the hierarchy
- Wider: target is a broader concept
- Equal, Narrower, Inexact: don’t use these match types
- Unmatched: don't leave anything as unmatched, instead find in a wider term and flag it (you can also write a comment) or ask the group for advice

Priority order, as seen in Figure A1.:

- Source concept is semantically one term: equivalent 1 concept 🡪 wider 1 concept
- Source concept is semantically several terms in one: equivalent 1 concept 🡪 equivalent 2-more concepts 🡪 wider 1/more concepts

**2.2.4 Documentation of issues**

If after mapping you're not sure if this is the right match, flag the code instead of approving it and be sure to write your thoughts in the comment section.

**2.2.5 Validation, conflict resolution**

The flagged matches are reviewed by the mapping core team. Approximately 5% of the codes are mapped by 2 or 3 mappers and the matches are compared with each other.

The core team also reviews the approved matches, to filter out as many of the mistakes as we can.

**3. After mapping**

**3.1 Quality review**

After the mapping with OHDSI’s Achilles tool we are to checking the data quality after the mapping.

Besides that, we discuss the lessons learned, what could have been better, what are the challenges for the future.

**3.2 Release final map**

Final map format

| Source ID | Source description | Target concept ID | Target description | Match type |
| --- | --- | --- | --- | --- |
|  |  |  |  |  |
|  |  |  |  |  |
|  |  |  |  |  |

**3.3. Maintenance**

Maintenance is needed as all the used code systems evolve and change with time.

**4. References**

1. Informatics, O.H.D.S.a. *Usagi usage - Auto Mapped*. [cited 2023 Jan 11]; Available from: <http://ohdsi.github.io/Usagi/usage.html#Auto_Mapped>.

2. DeepL. *Translator*. [cited 2023 Jan 11]; Available from: <https://www.deepl.com/translator>.

3. Center, N.H.S. *Pulvita Health Data Warehouse*. [cited 2021 May 14]; Available from: <https://prod.eadatlap.hu/>.

4. Resources, M.o.N. *9/2012 (II. 28.) NEFMI Decree*. [cited 2023 Jan 11]; Available from: <https://njt.hu/jogszabaly/2012-9-20-2V>.

5. Informatics, O.H.D.S.a. *Mapping of Concepts*. [cited 2023 Jan 11]; Available from: <https://www.ohdsi.org/web/wiki/doku.php?id=documentation:vocabulary:mapping>.

6. Alapkezelő, N.E. *Szabálykönyv a járóbeteg-szakellátás tevékenységi kódlistájának alkalmazásáról*. [cited 2023 1 February]; Available from: <http://finanszirozas.neak.gov.hu/szabalykonyv/index.asp>.

7. Alapkezelő, N.E. *Fekvőbeteg-szakellátási kézikönyv*. [cited 2023 1 February]; Available from: <http://www.neak.gov.hu/felso_menu/szakmai_oldalak/gyogyito_megeleozo_ellatas/szabalykonyvek/besorolo.html>.
